# Two-Step Nucleation and Amorphization of Carbamazepine Using a Micro-Droplet Precipitation System

**DOI:** 10.3390/pharmaceutics17081035

**Published:** 2025-08-09

**Authors:** Xiaoling Zhu, Cheongcheon Lee, Ju Hyun Park, Eun Min Go, Suha Cho, Jonghwi Lee, Sang Kyu Kwak, Jaehyeong Bae, Tae Seok Seo

**Affiliations:** 1 Department of Chemical Engineering (Integrated Engineering Program), College of Engineering, Kyung Hee University, 1732, Deogyeong-daero, Giheung-gu, Yongin-si 17104, Republic of Korea; zxl@khu.ac.kr (X.Z.); suha0116@khu.ac.kr (S.C.); 2 Department of Chemical Engineering and Materials Science, Chung-Ang University, 84 Heukseok-ro, Dongjak-gu, Seoul 06974, Republic of Korea; lcc612@cau.ac.kr (C.L.); jong@cau.ac.kr (J.L.); 3 Electrode Development Team, Samsung SDI Co., Ltd. R&D Center, 130 Samsung-ro, Yeongtong-gu, Suwon 16678, Republic of Korea; bjh814@unist.ac.kr; 4 Corning Technology Center Korea, Corning Precision Materials Co., Ltd., 212 Tangjeong-ro, Asan 31454, Republic of Korea; ghrtlql@unist.ac.kr; 5 Department of Chemical and Biological Engineering, Korea University, 145 Anam-ro, Seongbuk-gu, Seoul 02841, Republic of Korea; skkwak@korea.ac.kr

**Keywords:** carbamazepine, two-step nucleation, amorphization, liquid–liquid phase transition, intermediate phase

## Abstract

**Objectives**: Transforming poorly soluble crystalline drugs into their amorphous form is a well-established strategy in pharmaceutical science to enhance their solubility and improve their clinical efficacy. However, developing amorphous forms of organic drugs for pharmaceutical applications presents significant technical hurdles due to the lack of suitable analytical tools for the amorphization process. Carbamazepine is a crystalline BCS class II drug commonly used for epilepsy and trigeminal neuralgia, whose clinical efficacy is compromised by its low solubility and slow dissolution. Therefore, this study focuses on investigating the amorphization of carbamazepine to enhance its solubility by using a micro-droplet precipitation system. **Methods**: These micro-droplets serve as individual reactors, enabling homogeneous nucleation for precipitation of carbamazepine. During crystallization, carbamazepine undergoes an intermediate liquid–liquid phase transition characteristic of two-step nucleation. By varying the solvent’s composition (methanol/water), we characterized the kinetics and stability of the intermediate liquid phase under various conditions. **Results**: Our results indicate that carbamazepine can undergo either a one-step liquid-to-amorphous-solid phase transition or a two-step liquid-to-crystalline-solid phase transition. Notably, both transitions pass through a liquid-to-dense-liquid phase separation process starting from the supersaturated solution, where the generated intermediate phases exhibit different sizes and numbers that are influenced by the solvent and its concentration. **Conclusions**: Our findings not only elucidate the mechanism underlying the carbamazepine phase transition but also propose a novel method for studying the amorphous process, which could be broadly applicable to other poorly soluble pharmaceutical compounds and may be helpful to amorphous formulations production, potentially offering significant improvements in drug efficacy and patient compliance.

## 1. Introduction

Developing effective pharmaceuticals requires overcoming numerous challenges, particularly in drug solubility and bioavailability [1,2,3,4]. The oral bioavailability of a drug is determined by a number of properties, including the drug dissolution rate, its solubility, intestinal permeability, and pre-systemic metabolism. Therefore, the dissolution of solid dosage forms—tablets, capsules, and powders for oral applications—is of fundamental importance for bioavailability; this is because only dissolved drug molecules are able to diffuse through living tissue and improve drug absorption from the gastrointestinal tract. Consequently, the dissolution process often becomes the rate-limiting step in systemic drug delivery from solids [5,6].

The drug molecules characterized by low solubility or slow dissolution rates are particularly challenging to use effectively in clinical settings [7,8,9]. Compared to crystalline drugs, amorphous forms exhibit enhanced dissolution rates and higher apparent solubility. However, most amorphous drugs have poor physical stability and tend to recrystallize [10]. Although the physical instability of amorphous pharmaceuticals is a well-known limitation, extensive research has already been devoted to improving their stability, such as through the formulation of amorphous solid dispersions with polymers or the development of co-amorphous systems [11,12]. Therefore, stability concerns should not preclude further investigation into amorphous drug candidates. In this case, for those poorly soluble crystalline drugs used clinically, the transition from the crystalline state to the amorphous state represents a well-established pharmaceutical approach for solubility improvement. However, developing amorphous forms of organic drugs for pharmaceutical applications presents significant technical hurdles due to the lack of suitable analytical tools for amorphization. Therefore, it is critically important to identify a suitable analytical platform for studying the amorphous process during the crystallization of drugs.

**Figure 1:**
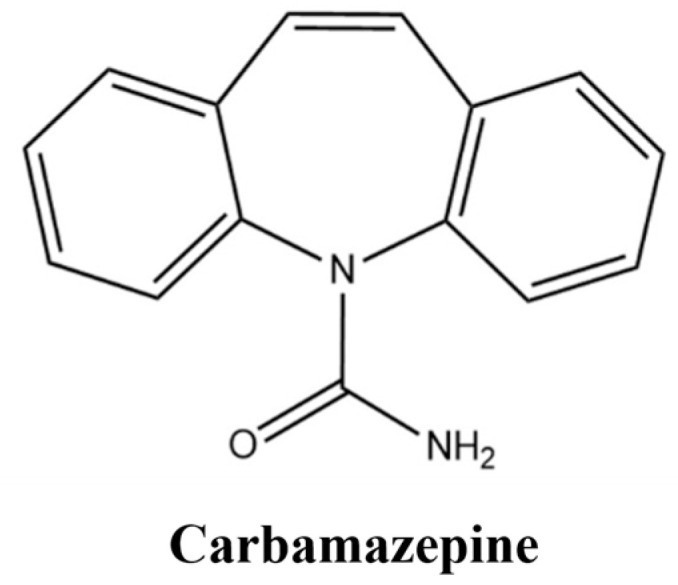
The chemical structure of carbamazepine.

Carbamazepine (CBZ) is a typical crystalline BCS class II model compound (MW = 236.27 g/mol, T_m_ = 192 °C, pka_1_ ≈ 1, pka_2_ ≈ 13.9, and LogP = 2.77) that is often involved in formulating readily usable medicines (Figure 1) [13,14,15,16,17]. Carbamazepine is a well-established antiepileptic drug widely prescribed for the treatment of various types of seizures and pain, including epilepsy, trigeminal neuralgia, bipolar disorder, and certain neuropathic pain syndromes. Despite its clinical importance, the pharmacological application of carbamazepine is hindered by its physicochemical properties, notably its low aqueous solubility (<238.64 μg/mL), which is independent of the pH of the medium, and its slow dissolution rate [18]. And these limitations stem from the drug’s hydrophobic chemical structure and its crystalline structures (triclinic form I, trigonal form II, monoclinic form III, monoclinic form IV, and orthorhombic form V) with various solubility parameters [19,20,21,22]. In addition, crystals are thermodynamically more stable as the solute molecules are orderly bound with stronger intermolecular forces. Using amorphous formulations or vitrification [23,24,25,26] is a promising strategy to address challenges faced by crystalline drugs like carbamazepine. The increased solubility not only improves systemic bioavailability but may also lower the administered dose, which could enhance medication adherence in patients [27,28,29,30].

Carbamazepine dihydrate is the primary crystalline form of carbamazepine when it is in the presence of water, an aqueous solution, or wet solvents such as bench ethanol. Broadhurst et al. captured the earliest stages of crystallization of carbamazepine dihydrate using a combination of cyro-transmission electron microscopy (cryoTEM) and three-dimensional electron diffraction (3D ED), which revealed not only short-lived polymorphs II, III, IV of carbamazepine but also the non-classical mechanism of crystal growth that begins with the formation of an initial film which separates into droplet-like structures, and the coalescence of these droplet-like structures then signals the next stage of crystal growth and development. The absence of Bragg reflections in the diffraction images of these droplet-like particles showed that they were amorphous and that the sample had been captured at a pre-crystallization stage [31]. This provides key guidance for research on the amorphous form of carbamazepine. However, the transition mechanism from droplet-like structures to crystalline phases remains unclear. And whether CBZ-II, III, and IV develop classically or non-classically has yet to be established [31]. In addition, the choice of solvent and its influence on the nucleation of CBZ have been investigated for a few polymorphs, but no systematic studies have been conducted so far [32].

Herein, to establish a suitable analytical platform for studying the amorphous process during the crystallization of drugs and capturing the early-stage crystallization mechanisms of carbamazepine, we present a novel micro-droplet precipitation system. This precipitation technique utilizes hundreds of micron-sized droplets as miniature reactors, which serve as a unique platform for conducting statistical analyses of carbamazepine phase transitions in environments devoid of impurities. Microfluidic technology has undergone rapid advancements in recent decades, finding extensive applications across life science disciplines. The droplet microfluidic platform is a type of microfluidic chip, and it has many advantages, such as a separate compartment, low consumption, good repeatability, and so on [33,34]. Importantly, this precipitation approach facilitates detailed observation of the nucleation process of the homogeneous phase that leads to crystal formation, as well as distinctive liquid-to-dense-liquid phase transitions that result in the formation of amorphous dense liquid clusters (ADLCs). The ADLC is a concept of the intermediate phase (or pre-nucleation clusters) of the two-step nucleation theory in the early stage of crystallization. During these transitions, the ADLCs demonstrate a transitional and/or terminal phase, offering significant insights into drug formulation and a broader understanding of amorphous organic drugs.

## 2. Materials and Methods

### 2.1. Materials

Polydimethylsiloxane (PDMS, Sigma Aldrich, Seoul, Korea), Fluorient FC-40 (Sigma Aldrich, Seoul, Korea), 008-Fluorosurfactant (RAN Biotech, MA, USA), glass wafers (iNexus, Inc., Gyeonggi-do, Korea), ethanol (Sigma Aldrich, Seoul, Korea), DI water, and carbamazepine (Sigma Aldrich, Seoul, Korea, mass purity ≥ 98%) were used.

### 2.2. Instruments and Method

#### 2.2.1. Microfluidic Chip Fabrication

The microfluidic droplet device was fabricated by conventional soft lithography using polydimethylsiloxane (PDMS) with a channel depth of 100 μm [35]. The PDMS part, which contained a continuous phase inlet, a dispersed phase inlet, a flow focusing zone, a tortuous mixer, and an outlet, was bonded with glass wafer counterparts after ozone treatment. The channel was thoroughly coated with aquapel for 10 s and blown by nitrogen gas. Then, the channels are incubated with FC-40 oil for 15 min prior to fluidic experiments.

Carbamazepine solutions with varying concentrations and ratios of methanol and water were injected. The generated droplets were collected onto the square cover glass (18 mm × 18 mm) containing 200 μL of FC-40 on the surface using a pipette. Then, the cover glass was placed on the stage of the polarized microscope (Nikon Eclipse TE2000-U, Daejeon, Korea) to observe the change process during CBZ crystallization.

#### 2.2.2. Carbamazepine Solution Preparation

The carbamazepine solutions were prepared according to the formulation described in Table 1. A saturated carbamazepine solution was prepared by adding an excess amount of carbamazepine to methanol and stirring it for 1 h to let it reach a saturated concentration at room temperature.

#### 2.2.3. Statistical Analysis of Phase Transitions

For the high-throughput statistical analysis of droplets, 50~100 droplets generated by the droplet chip were placed on the microscope and recorded. The size of the droplets was measured to calculate the increased concentration of the droplet by evaporation. The size and the number of the dense liquid clusters were analyzed to anticipate the carbamazepine concentration in the dense liquid clusters. The image was analyzed using the open-source software Image-J (version 1.54g).

#### 2.2.4. MD Simulation

To describe the interaction between carbamazepine and methanol molecules, all molecular dynamics (MD) simulations were performed using the COMPASS (condensed-phase optimized molecular potentials for atomistic simulation studies) forcefield [36,37] under the isobaric–isothermal (i.e., NPT) ensemble, where N is the number of atoms, P is the pressure, and T is the temperature. Atom types are defined based on chemical intuition. The pressure and temperature were controlled at 0.0001 GPa and 298 K using the Berendsen barostat and the Nosé–Hoover–Langevin (NHL) thermostat, respectively [38,39]. The time step for all MD simulations was set to 1 fs.

#### 2.2.5. CGMD Simulation

In order to model the carbamazepine clusters in the solvents, coarse-grained molecular dynamics (CGMD) simulations were performed. The carbamazepine molecule was modeled with a MARTINI force field [40]; the MARTINI model relies on an effective four-to-one mapping scheme, which means that on average four heavy atoms plus the corresponding hydrogen atoms of the organic molecules are represented by a single interaction site (CGbead), and the CG bead types are shown in Figure S7. For the simulation of carbamazepine clusters in the methanol and methanol–water mixture solvents, 32 carbamazepine clusters which had a diameter of 5 nm were introduced into a box with dimensions of 20 × 20 × 20 nm^3^. The mole ratio of carbamazepine to the solvents was 1 to 6. NPT (i.e., isothermal–isobaric) simulations were conducted at 298 K for 100 ns. The time step was 10 fs, and the cutoff radius for van der Waals interactions was 1.2 nm. A velocity-rescaling thermostat and a Parrinello–Rahman barostat were used for the isothermal–isobaric state, and the particle mesh Ewald method was used to treat Coulomb interactions. The RMSD of the carbamazepine clusters was obtained by analyzing the trajectories during the initial 20 ns of the simulation. CGMD simulations were conducted using the GROMACS 2016 package [41].

#### 2.2.6. X-Ray Powder Diffraction (XRPD)

XRD analysis of CBZ samples: The structural properties of the crystals were characterized with an X-ray diffractometer (Smartlab, Rigaku, Tokyo, Japan) equipped with a Cu Kα1 X-ray source. X-ray diffraction patterns were collected over angles ranging from 5° to 90°, with intervals of 0.01° and a scan rate of 5° per minute.

## 3. Results and Discussion

As mentioned previously, carbamazepine has low solubility in water, but it dissolves well in methanol (64 mg/mL) [17,42]. Thus, industries rely on methanol to dissolve and precipitate carbamazepine during production to obtain the desired crystal form. To match with the industrial process, we used a microfluidic device to produce uniform-microsize methanol-in-fluorinated oil droplets which contained carbamazepine. Particularly, fluorinated oil with a surfactant was used on behalf of hydrocarbon oils to compensate for the low surface tension of methanol (22.50 mN/m at 20 °C). Since methanol and water have a lower density than fluorinated oil, the droplets float and have an open interface with air, providing a channel for the solvent to evaporate. During this process, droplets shrunk, and the concentration increased accordingly. Droplet precipitators were observed and analyzed under a microscope by placing the carbamazepine/methanol droplets onto a square cover glass filled with fluorinated oil (Figure S1). In this setup, volatile methanol evaporates through the methanol/air interface during the whole process. This droplet-based precipitator is beneficial for analyzing phase transitions or crystallization due to the following reasons: 1. The droplet system does not have a solid interface that can act as nuclei, enabling homogeneous precipitation. 2. Only a small number of drugs are required for the experiment, making it good for studying expensive drugs or molecules that have low yields. 3. High-throughput screening is possible; 4. An increase in concentration over supersaturation can be processed by continuous methanol evaporation and monitored by tracking the size of the droplet.

**Figure 2:**
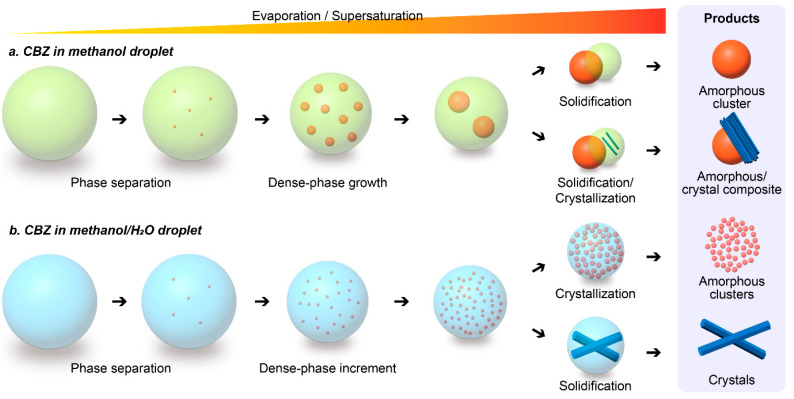
A schematic illustration depicts the various forms of carbamazepine (CBZ) in micro-droplets. In pure methanol droplets, carbamazepine forms either a large dense liquid cluster or a Janus-type cluster, depending on the concentration. Conversely, in a methanol/water mixture, carbamazepine forms many small dense liquid clusters or crystals by varying the ratio of methanol to water.

**Figure 3:**
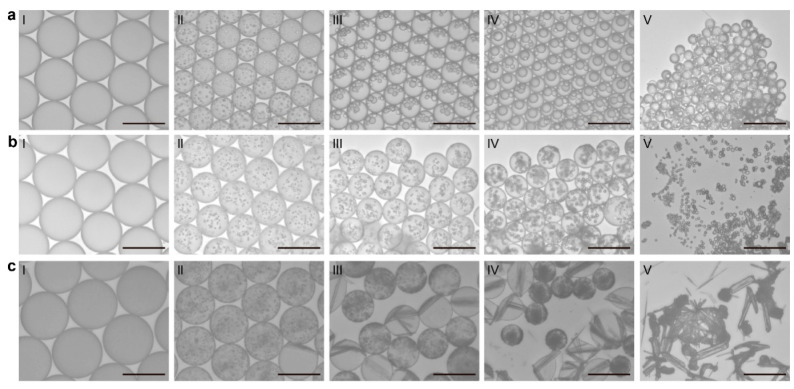
Formation of the dense liquid clusters from 3 mg/mL carbamazepine: (**a**) droplets in pure methanol; (**b**) droplets in the methanol/water mixture (90:10); (**c**) droplets in the methanol/water mixture (70:30).

**Figure 4:**
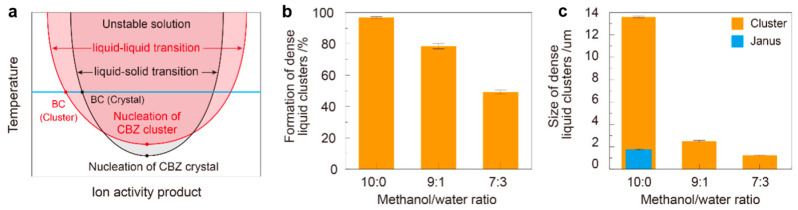
(**a**) Schematic phase diagram of the carbamazepine–methanol system. Two binodal lines are shown for the liquid–liquid phase transition and the liquid–solid phase transition. The blue line represents an increase in the ion activity product at a constant temperature. BC (Cluster): binodal line for the carbamazepine cluster. BC (Crystal): binodal line for the carbamazepine crystal. (**b**) Ratio of cluster formation to crystallization in droplets. (**c**) Diameter of the dense liquid clusters in different solvent conditions. Scale bar (**a**–**c**): 50 μm.

In this droplet precipitation system, we produced a series of solid forms of carbamazepine from a methanol or methanol/water mixture with various concentrations, where we successfully managed to produce either the dense liquid cluster form or the crystal form of carbamazepine by varying the precipitation condition in the droplets (Figure 2). As an example, the droplets containing 3 mg/mL of carbamazepine in pure methanol were analyzed (Figure 3(aI)). As the solvent evaporates to increase the concentration of the droplet to supersaturation, we found a clear liquid-to-liquid phase separation with nucleation and growth characteristics (comparable to spinodal decomposition) in the droplet, which was not expected, as carbamazepine in bulk production normally gives needle-shaped solid crystals, as shown in Figure 3(cIII). To clarify the difference between the liquid–liquid phase separation with nucleation and solid crystal nucleation characteristics, we call the former phenomena liquid-phase nucleation. Liquid-phase nucleation appeared at 5.59 mg/mL, which is a lower concentration than that observed during crystal supersaturation [17]. As the physical/chemical state of the dense liquid cluster phase is different from the crystal, it is obvious that the phase diagram of the carbamazepine solution to the dense liquid cluster is differentiated from the crystal (Figure 4a).

The observed evolution of the dense liquid cluster of carbamazepine is very similar to the pre-nucleation droplet-like structure observed in Broadhurst et al.’s study and also similar to cluster formation in many other materials, including calcium carbonate, proteins, and other organic molecules [31,43,44,45,46,47]. The reproducibility of these phenomena across numerous droplets confirms that the experiment is not stochastic [48]. After the initial appearance, both the size and the number of the dense liquid clusters of carbamazepine continuously increased. During this process, crystal nucleation was absent. Instead, the cluster grew and coalesced into a larger dense liquid cluster (dia. 13.5 μm; Figure 3(aIII–aV), and Supporting Video S1). This was the largest dense liquid cluster reported to date. The dense liquid phase exhibited liquid-like characteristics, maintaining a spherical shape to reduce the surface tension, suggesting that the cluster contains a certain amount of methanol (Figure S2) [49]. So if the solution maintains a saturated-to-equilibrate state with the dense liquid cluster, regardless of the size effect, the concentration of the cluster at the final stage was calculated to be 0.69 mg/mL, which is a molecular ratio of methanol to carbamazepine of 3.88:1. At the final stage, we observed the formation of some crystals that may contain a mixture of forms II, III, IV in the liquid phase adjacent to the dense liquid phase, while the dense liquid phase maintained its structure (Figure 3(aV)). This behavior is distinct from previously reported pre-nucleation clusters, which diminish with crystal formation, indicating unique properties of the carbamazepine/methanol system compared to other systems. For example, the carbamazepine dense liquid cluster is distinct in its stability compared to the pre-nucleation clusters of calcium carbonate, which only temporarily form under in situ TEM or cryo-TEM conditions [46,47]. Under TEM, the CaCO_3_ dense liquid cluster existed temporarily, with its growth limited to the micron scale by subsequent crystal nucleation. However, the stability of CaCO_3_ dense liquid clusters was not clearly studied, as their formation was influenced by solid interfaces and the intensity of the electron beam. But in our droplet precipitation system, the growth and stability of the clusters are mainly governed by the surface energy of the clusters as they are free from such external factors. And thermodynamically, larger clusters are more stable than smaller ones due to lower surface free energy.

To understand the effect of the solvent, we added 10% *v/v* of water, which acts as an antisolvent compared to methanol, to the droplet in the same experimental setup. Initially, small dense liquid clusters formed in a manner similar to those in pure methanol. However, these clusters did not grow as much as they did in pure methanol; instead, their number increased continuously until the solvent had completely evaporated (Figure 3(bII–bV) and Supporting Video S2). The clusters formed in the presence of water were smaller and did not merge, remaining small (Figure 3(bV)). One plausible reason for the limited growth and segregation of the dense liquid clusters could be the distinct interaction of carbamazepine with methanol and water molecules, as described by their solubility parameters. The total solubility of carbamazepine is 24 MPa^0.5^, which is close to 28 MPa^0.5^ for methanol but far from 47.8 MPa^0.5^ for water [50]. Solubility parameters derived from the cohesive energy density (CED) of molecules indicate that molecules with similar CEDs tend to associate. Therefore, carbamazepine interacts more strongly with methanol through hydrogen bonds than with water; methanol can dissolve more carbamazepine molecules than water. As a result, carbamazepine segregates water and methanol locally, leading to the formation of dense liquid clusters. In this condition, we observed a lower likelihood of carbamazepine crystal formation—only a few droplets produced crystals, while most of the small dense clusters were vitrified. This is because water and methanol are miscible, and the surface of the cluster is partially surrounded by a water layer, which acts as a barrier for the mass transport of carbamazepine and prevents the clusters from merging. The pathways of diffusion and aggregation are isolated so that the crystalline lattice structure cannot be built up easily.

We further increased the water content in the droplets to a methanol ratio of 70:30 while maintaining the same carbamazepine concentration (Figure 3c). The precipitation process was similar to that in droplets with a 90:10 ratio; however, the size of the dense liquid clusters became smaller, and the number of clusters increased. Notably, after many clusters formed, crystal nucleation of carbamazepine occurred in most of the droplets (Figure 3c and Supporting Video S3). Similar phenomena were observed at the increased concentration of 9 mg/mL (Figure S3). This nucleation could be initiated by the reduced space for the pure methanol domain due to the increased amount of water. Additionally, the solubility limit of carbamazepine in water is much lower than that in methanol, leading to a phase transition in the water-rich domain. Once crystals nucleated and grew, the surrounding carbamazepine clusters dissolved into the solvent and diffused toward the crystals, which have lower thermodynamic energy. Thus, by adjusting the solvent’s composition, the phase transition of carbamazepine can be directed toward either amorphous or crystalline forms. Our findings reveal a significant disparity in the energy barriers for cluster formation and crystal nucleation. The energy level of the dense liquid cluster surpasses that of crystallization, as evidenced by the dissolution of clusters during crystallization (Figure 4a). This kinetic control of cluster formation and thermodynamic control of crystal nucleation represent a novel understanding in the field.

In our experiments, a small amount of water inhibited the growth and coalescence of dense liquid clusters. Only a large amount of the antisolvent facilitated crystallization (Figure 4b,c). Although carbamazepine and water interact poorly with each other, both are favorable to methanol, creating an interesting interrelationship at the cluster interface. Water molecules, which are attracted by methanol, form a barrier to carbamazepine diffusion, thereby reducing the size of the clusters as the water content increases. Moreover, increased water content reduces the solubility of carbamazepine in the droplet, improving the degree of supersaturation of the solution and facilitating crystal nucleation. This suggests that the thermodynamic conditions for cluster formation and crystal nucleation can be independent. Additionally, small clusters are unstable due to their large interfacial energy; therefore once crystals form, the clusters dissolve and diffuse toward the growing crystals. Thus, the presence of an antisolvent plays a crucial role in determining whether carbamazepine precipitates into crystals or dense liquid clusters.

**Figure 5:**
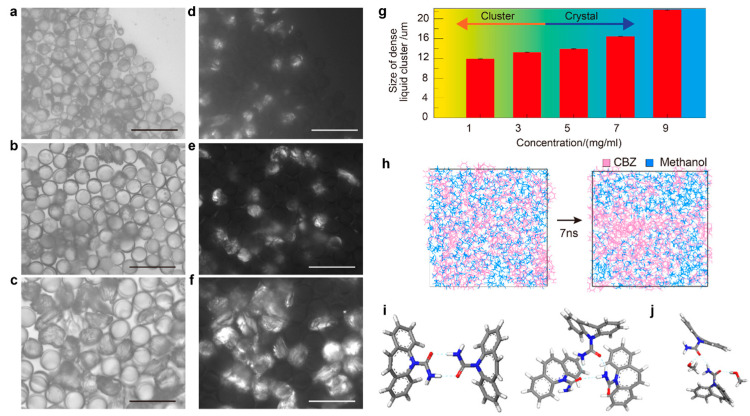
Dense liquid clusters from different initial concentrations in pure methanol droplets. (**a**–**c**) Microscopic image of the formed dense liquid clusters in pure methanol droplets with concentrations of 3, 7, and 9 mg/mL. (**d**–**f**) Polarized microscopic image of (**a**–**c**). Scale bar (**a**–**f**): 50 μm. (**g**) Size of the dense liquid clusters across varying concentrations and concentration-dependent partial crystallization. (**h**) Initial bulk system and equilibrium state (1 atm, 298 K) of carbamazepine. Methanol clusters with a ratio of 100:600 represent a carbamazepine solution with a concentration of 3 mg/mL. Carbamazepine and methanol molecules are shown in pink and sky blue, respectively. The most stable configurations (**i**) of carbamazepine and (**j**) carbamazepine in methanol from MD simulations. Gray, blue, red, and white represent carbon, nitrogen, oxygen, and hydrogen, respectively.

Our droplet reactor provides a statistical analysis platform for a large number of experiments in a reproducible manner. We hypothesized that the size of the dense liquid cluster and the initial concentration of carbamazepine in droplets would have a linear relationship since the concentration should remain the same during the process based on the classical Gibbs phase separation theory of the mixture. To confirm, we produced a series of dense liquid clusters by varying the initial concentrations to 3, 5, 7, and 9 mg/mL (Figure 5a–f). The results show that the size of the cluster and the initial concentration have a linear relationship, which indicates that the cluster may undergo solvatation, and there is an optimal amount of methanol in the cluster (Figure 5g). Specifically, based on the initial concentration, the concentration of the cluster could be 2.42~3.88 mg/mL.

To understand the metastability of the carbamazepine/methanol cluster, intermolecular packing in the cluster was calculated using MD simulations based on the experimental condition. (see Section 2, Figure 5h–j and Figure S4). First, we set two different ratios of clusters: one has a carbamazepine-to-methanol ratio of 100:600, and the other has a carbamazepine-to-methanol ratio of 100:1200. When the system reached equilibrium, the calculated density was 1.01 g/cm^3^ for the 100:600 ratio and 0.94 g/cm^3^ for the 100:1200 ratio, and the trend aligns with the carbamazepine-to-methanol ratio in the cluster. In the two systems, we found that there are two stable carbamazepine-to-carbamazepine structures (Figure 5i). The stable structure originated from the hydrogen bonding of oxygen and hydrogen atoms in carbamazepine. In addition, the systems also have a stable carbamazepine-to-methanol structure. This is caused by the hydrogen bonding of oxygen and hydrogen atoms in carbamazepine and methanol molecules (Figure 5j). On the other hand, we have also calculated the equilibrium state, starting from carbamazepine packed in the crystalline structure of form II of carbamazepine molecules surrounded by methanol molecules. When the system is equilibrated, the crystalline structure of carbamazepine becomes amorphous, which shows the metastability of dense liquid clusters, even with carbamazepine in its crystalline form. With the same carbamazepine-to-methanol ratio, the calculated equilibrium densities were 1.00 g/cm^3^ and 0.93 g/m^3^, which are similar to the calculation starting from the random distribution. Moreover, in the 1:6 ratio of the cluster, carbamazepine exists by maintaining the layered structure, while the 1:12 ratio of the cluster has a more random distribution of the molecules.

In recent years, the modified Gibbs phase separation theory suggests that the concentration of the phase nuclei can be varied, which is different from the classical Gibbs phase separation theory, where the concentration of the phase nuclei should be constant. The variation in the concentration may be caused by the different evaporation rates of the droplets, which are a crucial factor in the diffusion process as rapid evaporation increases the vortex in the droplets. An increased vortex can induce a variation in the concentration of the clusters as it influences the formation of the coupling of carbamazepine with methanol. In addition, dense liquid clusters were formed when the initial concentration was lower than 3 mg/mL, whereas the droplets from concentrations greater than 3 mg/mL formed Janus-type carbamazepine (half-crystal–half-cluster composite) (Supporting Video S4). Above 3 mg/mL, the volume of the solution becomes less than the cluster, and it covers only half of the cluster surface in high concentrations (Figure S3). In such conditions, there are three-phase contact lines, which include the cluster, methanol, and oil phases. These three-phase contact points can locally increase the concentration to achieve the supersaturation point for crystal nucleation. However, since the large size of the cluster did not undergo dissolution and transform into crystals during this process, it left the product of Janus-type carbamazepine.

**Figure 6:**
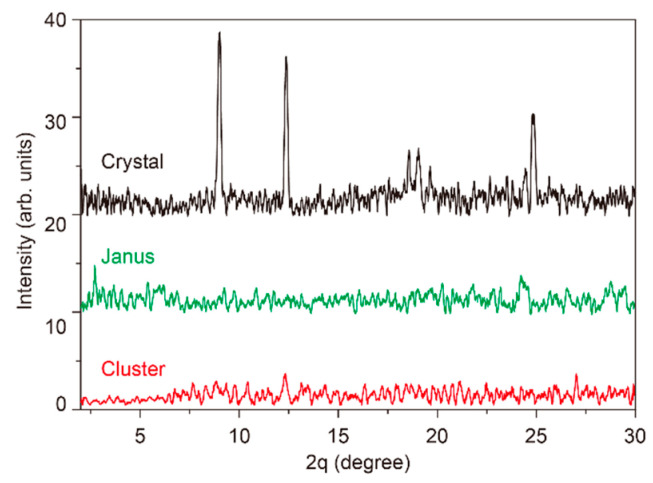
XRD analysis of crystalline carbamazepine, Janus-type carbamazepine, and a cluster form of carbamazepine.

X-ray diffraction was conducted to analyze the crystalline structure of the three different forms of carbamazepine. As shown in Figure 6, the needle-shaped crystals were identified as form A of carbamazepine with peaks near 8°, 12°, and 24°. However, the vitrified dense liquid clusters did not produce a clear peak in the XRD analysis, which shows that the vitrified clusters possess an amorphous nature. The notable thing is that Janus-type carbamazepine also does not produce a peak in XRD. This can be interpreted as the crystalline portion taking a minority of the product, or the crystal in the Janus phase is poorly arranged. Anyhow, both amorphous carbamazepine and Janus-type carbamazepine are expected to have higher dissolution rates compared to crystalline carbamazepine based on their crystallinity.

**Figure 7:**
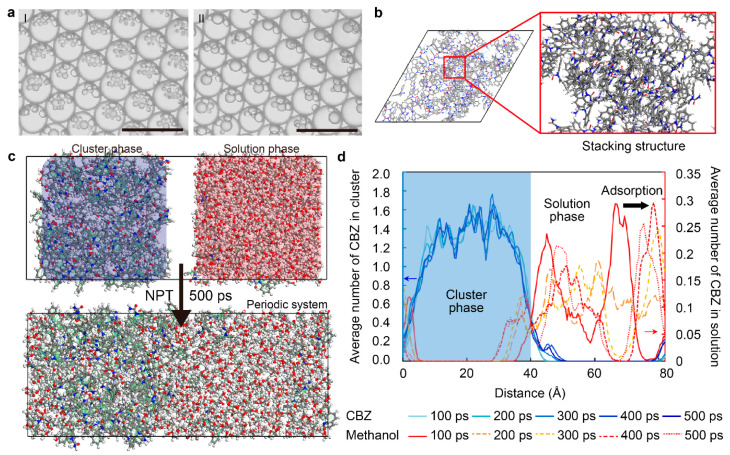
Physical properties of the dense liquid clusters. (**a**) Coalescence of the dense liquid clusters in droplets (condition of the solution: 3 mg/mL carbamazepine in pure methanol). (**b**) The stacking structure of carbamazepine clusters at a carbamazepine-to-methanol ratio of 1:6. The molecular structure diagram demonstrates that the clusters maintain consistent layers and exhibit a regular, orderly structure. (**c**) The result of NPT simulations of the methanol phase on carbamazepine phase II at the lowest surface energy. (**d**) A time-lapse NPT simulation showing the adsorption of carbamazepine from the methanol solution phase onto the cluster phase on the [2, −1, 0] surface of carbamazepine phase II. Over time, carbamazepine molecules in the methanol solution increasingly adhere to the stable surface, forming clusters. Bule arrow indicates CBZ density increase in cluster phase, black and red arrow indicates methanol adsorption toward solution phase. Gray, blue, red, and white represent carbon, nitrogen, oxygen, and hydrogen, respectively.

During the formation of dense liquid clusters, we found several unique physical properties. The first is coalescence among the clusters within the solution without antisolvents (Figure 7a and Supporting Video S5). The clusters tended to gather together, and intimate clusters coalesced, followed by their shape turning into a sphere. Their coalescence greatly resembles the behavior of the liquid droplets, which can be explained by the proportion of methanol in the dense liquid cluster. Coalescence would have proceeded to reduce their surface energy to form a stable form of dense liquid clusters. To understand coalescence, the interfacial structure between the cluster phase and the methanol solution phase was calculated using MD simulations (Figure 7b; details in Section 2). Due to the cluster phase being more stable than the solution phase, the carbamazepine in the methanol solution phase tends to adsorb to the interface (Figure 7c). To quantify the phenomena, the average number of carbamazepine molecules contained in each cluster and methanol solution phase is measured within the interfacial system. As the average number of cluster phases is maintained, the carbamazepine molecules in the methanol solution phase tend to move toward a cluster phase to progress in the phase transition (Figure 7d).

**Figure 8:**
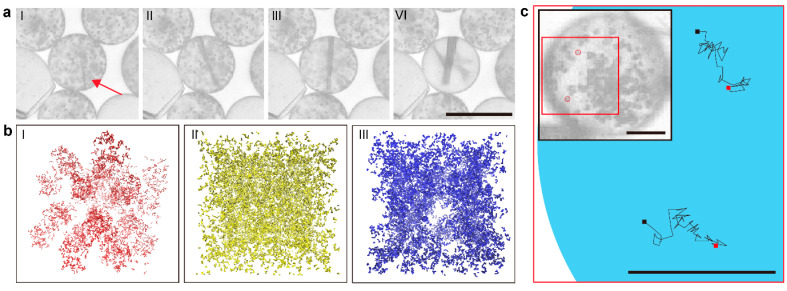
(**a**) Crystal nucleation followed by dissolution of the dense liquid clusters in droplets (formation condition: 3 mg/mL carbamazepine in the methanol/water mixture (70:30)). The red arrow indicates initial solid nucleation. (**b**) Comparison of carbamazepine clustering in two solvent environments: methanol alone and a methanol–water mixture (70:30 molar ratio) at a carbamazepine-to-solution ratio of 1:6. I: initial state, II: methanol alone, and III: methanol–water mixture. (**c**) Brownian motion of the dense liquid clusters in droplets (condition of the solution: 3 mg/mL carbamazepine in the methanol/water mixture (70:30)). High-quality images were extracted (5 frames per second) from recorded 10 s videos. Two clearly discriminable clusters (red circles in the red box) were selected for tracing. Scale bar (**a**, **b**): 50 μm. (**c**) Scale bar for the inset of (**c**): 10 μm.

The second is the dissolution of the small clusters followed by crystal nucleation in the droplet with the antisolvent (Figure 8a and Figure S5 and Supporting Videos S6 and S7). As the crystal nucleated and grew rapidly, small clusters started to dissolve from near the nucleation spot to the entire droplet. Such a finding agrees with the dissolution of amorphous calcium carbonate followed by crystallization [51]. The crystal needs to absorb the surrounding solute to continue its growth, so the solution near the growing crystals becomes unsaturated. At the same time, the reduced carbamazepine concentration in the solvent was complemented by dissolving the surrounding small dense liquid clusters to provide a solute for the solution. Small, dense liquid clusters are likely to dissolve due to their high surface energy, small size, and curvature. We suggest that this is the main reason why the dense liquid cluster fails to persist until the end of crystallization in a conventional experimental setup.

The small size of the carbamazepine cluster with the existence of water was further studied using CGMD simulations. The behavior of clusters with a diameter of 5 nm (Figure 8(bI)) was compared in methanol and methanol–water mixture solvents, respectively. Without water, the clusters coalesced and became a single cluster (Figure 8(bII)). However, when water existed, the clusters were aggregated more densely compared to the clusters without water (Figure 8(bIII)). In addition, while the carbamazepine molecules diffused from a cluster into methanol in the absence of water, diffusion was found to be less in the clusters in the methanol–water mixture, as shown in the RMSD of carbamazepine of each cluster (Figure S6). Therefore, small-sized carbamazepine clusters were expected to be better maintained in the presence of water due to the hydrophobic effect.

The third is the vigorous random movement of dense liquid clusters in the droplet, which is very similar to Brownian motion (Figure 8c and Supporting Video S8). This behavior was only observed in the presence of water, where the size of the clusters is about 1 μm. We studied a single cluster with a diameter of 20 nm where the cluster without water tends to diffuse, but the cluster tends to aggregate in the water. The root mean square distance of carbamazepine in the cluster clearly shows the different distributions of carbamazepine in each solution. Moreover, the cluster exhibits Brownian motion in the molecular simulation. The surface of the clusters has both carbamazepine and methanol molecules, where water would attract hydrophilic methanol and repel hydrophobic carbamazepine. Such selective interactions patterns and repulsion forces created vigorous random motion. In addition, evaporation also induces some internal flux via the Marangoni effect [51]. Therefore, dense liquid clusters should be classified as intermediates between liquids and solids as they exhibit both liquid-like and solid-like behaviors similar to liquid crystals.

**Figure 9:**
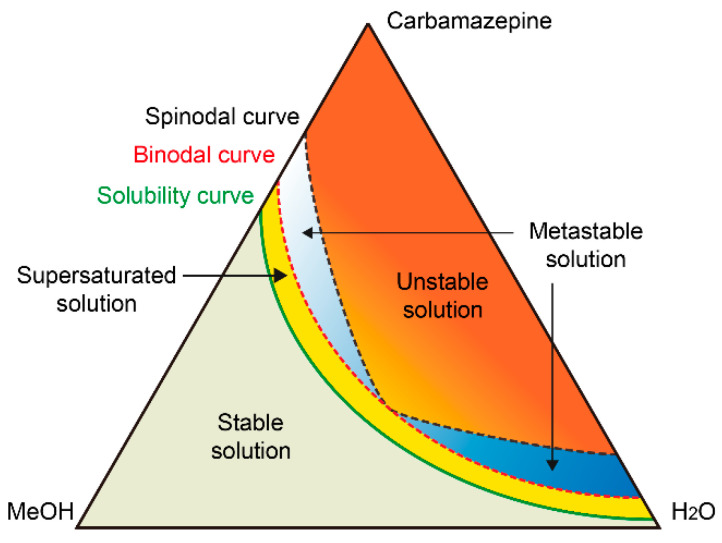
Schematic illustration of the phase diagram for the ternary carbamazepine–methanol–water system at room temperature. The blue gradient sections indicate the zones of phase transformations. (The deeper color indicates the higher likelihood of crystallization).

In the evaporating droplet system, the solution exceeds its solubility limit and undergoes liquid–liquid phase separation by means of binodal demixing, i.e., a nucleation and growth manner (Figure 9). The concentration of the supersaturated solution may be maintained in a certain value by continuous formation of dense liquid clusters. Thus, the condition of the solution depends on the progression from the appearance of the cluster to its transformation within the solution. Also, as the condition of the solution is close to that of water in the ternary-phase diagram, the likelihood of crystallization increases.

Dense-liquid clusters have been regarded as an intermediate phase during nucleation; therefore, new nucleation theories, including the multi-step nucleation theory and the multi-phase nucleation theory, have emerged, which contradict the classical nucleation theory developed by Gibbs [52,53,54,55]. However, the role of the dense liquid cluster was not clearly explained due to a lack of data regarding the concentration for thermodynamic studies, the limited cluster size, and a lack of experimental setups for genuine homogeneous precipitation [44,46,47]. Broadhurst et al.’s study attributed carbamazepine’s two-step nucleation mechanism to droplet coalescence phenomena, suggesting it as a precursor to the next phase, but their study lacked more detailed discussion [31]. From our result, we suggest crystal nucleation near the interface between the solution and clusters. Crystal nucleation could be induced by increasing the initial solute concentration with the existence of an antisolvent. At high concentrations without an antisolvent, the dense liquid cluster contains a low amount of methanol, which increases the solute concentration of the solution because of the pseudo-equilibrium between the dense liquid cluster and the solution. Thus, increased density fluctuations near the cluster induce crystal nucleation and increase the probability of nucleation. On the other hand, if an antisolvent was added, the vigorous motion of the dense liquid cluster would increase the structural fluctuation near the cluster, which facilitates crystal nucleation, and the surface of the newly formed crystalline particle may contain a large number of imperfections that would encourage further rapid crystalline growth.

## 4. Conclusions

In this work, we introduce a micro-droplet precipitation strategy that enables direct investigation of amorphous formation during drug crystallization and provides unprecedented insights into the early-stage nucleation mechanisms of carbamazepine (CBZ). Our findings not only reinforce the non-classical nucleation pathway previously proposed for CBZ but also offer compelling evidence to delineate the transition states underlying this process. While earlier studies suggest the potential emergence of alternative CBZ polymorphs (II, III, and IV) during the pre-nucleation phase [31], the complexity of these transitions remains insufficiently understood. Moving forward, we aim to (1) elucidate whether all CBZ polymorphs adhere to a two-step nucleation mechanism, (2) characterize the polymorphic assemblies governing distinct pre-nucleation stages, (3) assess the solubility and physical stability of amorphous CBZ produced via this approach once scalable synthesis is achieved, and (4) extend this methodology to other poorly soluble crystalline drugs. Collectively, this framework establishes a versatile platform for probing nucleation pathways and tailoring amorphous drug design.

In summary, as the crystal state of a drug greatly influences its therapeutic efficacy and administration method, our study provides significant insights into controlling and analyzing the crystallinity of carbamazepine using a micro-droplet precipitation system. We demonstrated that carbamazepine can transition between amorphous and crystalline forms by adjusting solvent compositions and concentrations within micro-droplets. This approach not only elucidates the phase transition mechanisms of carbamazepine but also offers a novel method for producing amorphous pharmaceutical formulations, which could also be broadly applicable to other poorly soluble drugs. The ability to modulate drug crystallinity through such controlled methods offers a possibility to the development of more effective drug forms, potentially enhancing drug solubility, bioavailability, and patient compliance. Our findings pave the way for future research in optimizing drug formulations to achieve better therapeutic outcomes.

## Figures and Tables

**Table 1:** Carbamazepine precipitation formulation for the droplet precipitator.

Carbamazepine Concentration (mg/mL)		1	3	5	7	9	3	3
Solution composition (*v*/*v*)	MeOH (%)	100	100	100	100	100	90	70
	Water (%)	0	0	0	0	0	10	30

## Data Availability

The data that support the findings of this study are available from the corresponding author upon reasonable request.

## Supplementary Materials

The following supporting information can be downloaded at https://www.mdpi.com/article/10.3390/pharmaceutics17081035/s1. Figure S1. Schematic illustration of evaporating droplets in fluorinated oil. The droplets float on the air/oil interface, and the solvent of the droplets evaporates into the air. Figure S2. Crystalline-dependent birefringence of carbamazepine. (a) Microscopic image of dense liquid clusters from 3mg/mL carbamazepine in pure methanol droplets. (b) Microscopic image of dense liquid clusters from 3 mg/mL carbamazepine in droplets from the methanol/water mixture (9:1). (c) Microscopic image of dense liquid clusters from 3 mg/mL carbamazepine in droplets from the methanol/water mixture (9:1). d–f are the polarized microscopic images of a–c, respectively. Only the crystals of carbamazepine showed a birefringence nature, whereas dense liquid clusters did not. Scale bar a–f: 50 µm. Figure S3. Formation of the dense liquid cluster in high concentrations. (a) Formation of dense liquid clusters from 9 mg/mL carbamazepine in pure methanol droplets. (b) Formation of dense liquid clusters from 9 mg/mL carbamazepine in droplets from the methanol/water mixture (9:1). (c) Formation of dense liquid clusters from 9 mg/mL carbamazepine in droplets from the methanol/water mixture (7:3). Scale bar a–c: 50 µm. Figure S4. (a) Packing model and NPT simulation (1 atm, 298 K) of CBZ. Methanol clusters with a ratio of 100:1200 represent the CBZ solution with a concentration of 9 mg/mL. (b) The comparison of the CBZ centroid to the methanol centroid at different molecular ratios. (c) Radial distribution function (RDF) graph comparison for CBZ clusters with CBZ-to-MeOH ratios of 1:6 and 1:12. Figure S5. Crystal nucleation followed by the dissolution of the dense liquid clusters in droplets. Figure S6. Root mean square deviation (RMSD) graph demonstrating the diffusion behavior of CBZ with and without the presence of water. Figure S7. All atoms (left) and the CG model (right) for the CBZ molecule. Table S1. Methanol-to-carbamazepine ratios in dense liquid clusters with different initial concentrations. Supporting Video S1. Whole process of dense liquid cluster formation from 3 mg/mL carbamazepine in pure methanol droplets. Supporting Video S2. Whole process of dense liquid cluster formation from 3 mg/mL carbamazepine in droplets from the methanol/water mixture (9:1). Supporting Video S3. Whole process of dense liquid cluster formation from 3 mg/mL carbamazepine in droplets from the methanol/water mixture (7:3). Supporting Video S4. Crystallization of carbamazepine during evaporation. Supporting Video S5. Coalescence of the dense liquid clusters inside the droplets. Supporting Video S6. Crystal nucleation followed by the dissolution of the dense liquid clusters in droplets. Supporting Video S7. Crystal nucleation followed by the dissolution of the dense liquid clusters in droplets with high concentrations. Supporting Video S8. Brownian motion of the clusters inside of a droplet.

## Abbreviations

The following abbreviations are used in this manuscript:

ADLCs	Amorphous dense liquid clusters
PDMS	Polydimethylsiloxane
MD	Molecular dynamics
NPT	Number of atoms, pressure, and temperature
CGMD	Coarse-grained molecular dynamics
CBZ	Carbamazepine
XRD	X-ray Diffraction
