# Supplementary material for: Two-Step Nucleation and Amorphization of Carbamazepine Using a Micro-Droplet Precipitation System
Source: Pharmaceutics. 2025 Aug 9;17(8):1035. doi: 10.3390/pharmaceutics17081035 (PMC12389563; doi:10.3390/pharmaceutics17081035)
Supplement: Supplementary file 1 [file pharmaceutics-17-01035-s001.zip › pharmaceutics-3781678-supplementary/CBZ X. Zhu Supplementary Information.pdf]

Supplementary Information

Two-step nucleation and amorphization of carbamazepine using a micro-droplet precipitation system

Xiaoling Zhu, Cheongcheon Li, Ju Hyun Park, Eun Min Go, Suha Cho, Jonghwi Lee, Sang Kyu Kwak, Jaehyeong Bae and Tae Seok Seo**

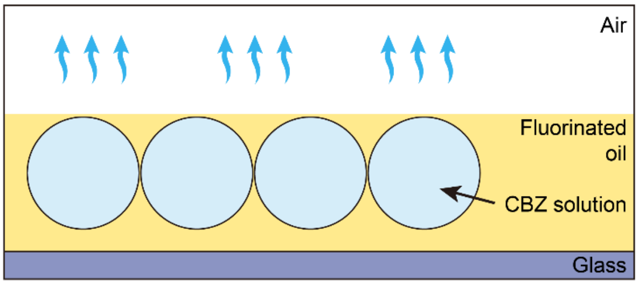

Figure S1. Schematic illustration of evaporating droplets in fluorinated oil. The droplets float on the air/oil interface, and the solvent of the droplets evaporates into the air.

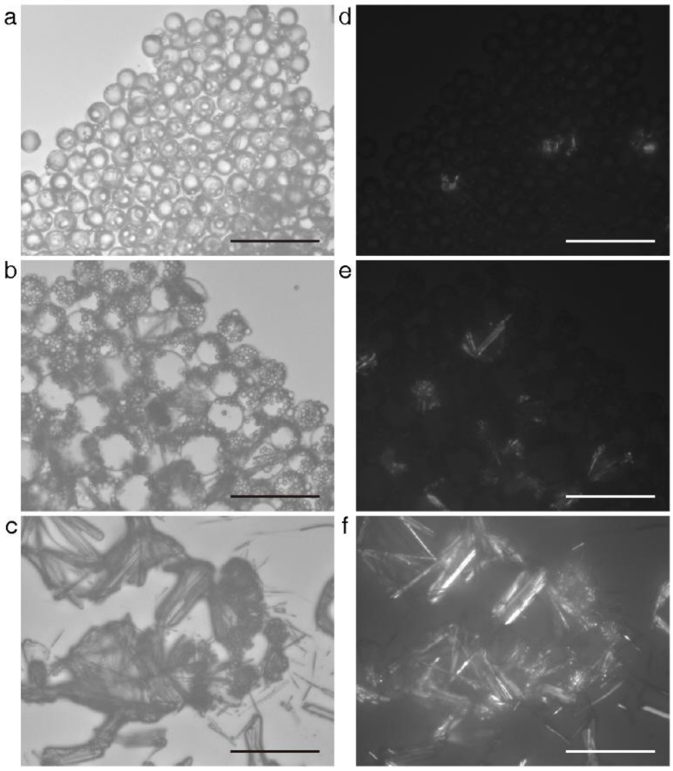

Figure S2 . Crystalline dependent birefringence of carbamazepine. **a** Microscopic image of dense liquid clusters from 3mg/ml carbamazepine in pure methanol droplets. **b** Microscopic image of dense liquid clusters from 3 mg/ml carbamazepine in the methanol: water mixture (9:1) droplets. **c** Microscopic image of dense liquid clusters from 3 mg/ml carbamazepine in the methanol: water mixture (9:1) droplets. **d-f** are the polarized microscopic images of a-c, respectively. Only the crystals of carbamazepine showed a birefringence nature, whereas dense liquid clusters did not. Scale bar, a-f: 50 µm.

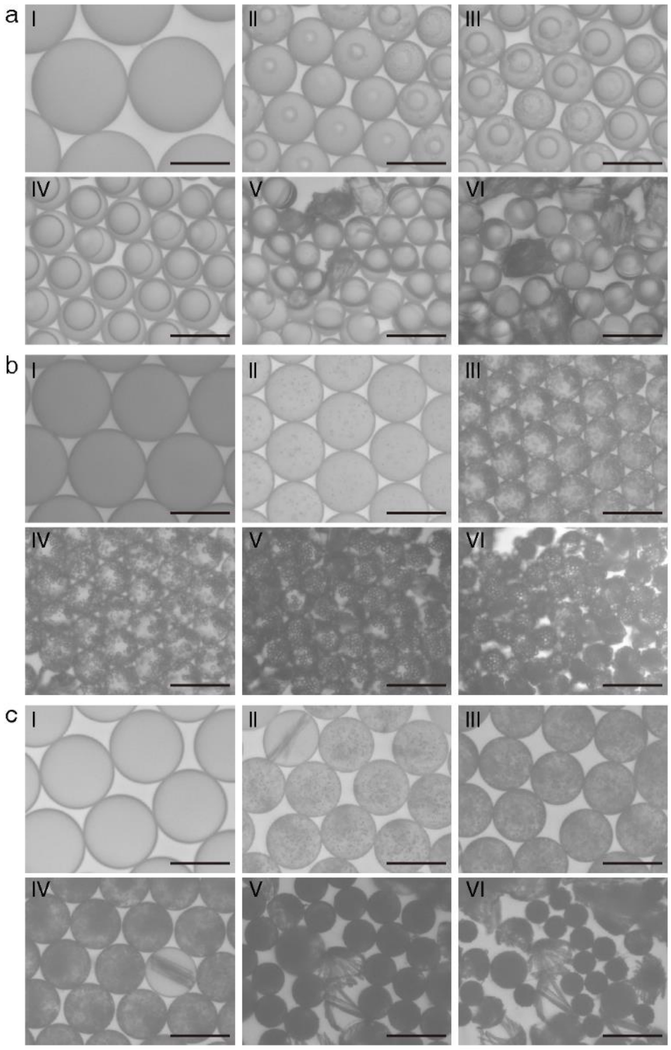

Figure S3. Formation of the dense liquid cluster in high concentration. a Formation of dense liquid clusters from 9 mg/ml carbamazepine in pure methanol droplets. **b** Formation of dense liquid clusters formation from 9 mg/ml carbamazepine in the methanol: water mixture (9:1) droplets. **c** Formation of dense liquid clusters formation from 9 mg/ml carbamazepine in the methanol: water mixture (7:3) droplets. Scale bar, **a-c**: 50 µm.

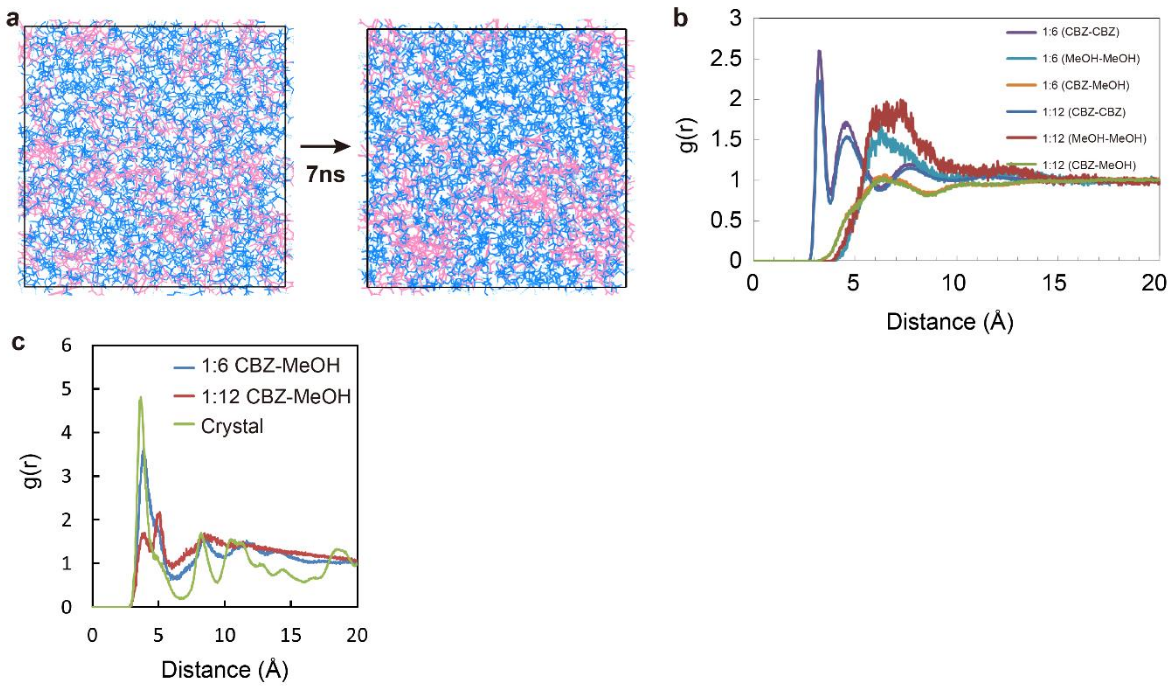

Figure S4. a Packing model and NPT simulation (1 atm, 298 K) of CBZ: methanol clusters with a ratio of 100:1200 represents CBZ solution of 9 mg/ml. **b** The comparison of CBZ centroid to methanol centroid at different molecular ratios. **c** Radial distribution function (RDF) graph comparison for CBZ clusters with CBZ to MeOH ratio of 1:6 and 1:12. The RDF graph for the 1:12 shows an amorphous distribution, indicated by the lack of distinct peaks. In contrast, the 1:6 concentration exhibits pronounced peaks, corresponding to a regular, layered structure as shown in the molecular structure diagram. This suggests that CBZ to methanol ratio of 1:6, the clusters maintain a stable arrangement similar to CBZ Form II, highlighted by the green peaks in the RDF graph, indicating a stable and orderly molecular configuration.

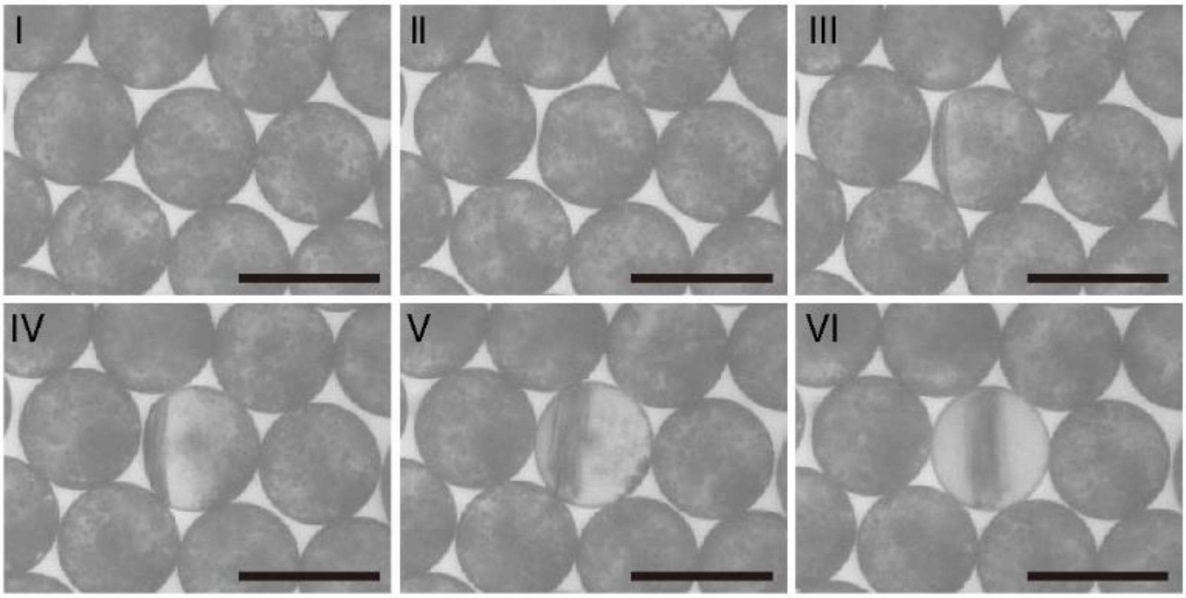

Figure S5. Crystal nucleation followed by the dissolution of the dense liquid clusters in droplets. The condition of the droplet was 9 mg/ml carbamazepine in methanol: water mixture (7:3) droplets.

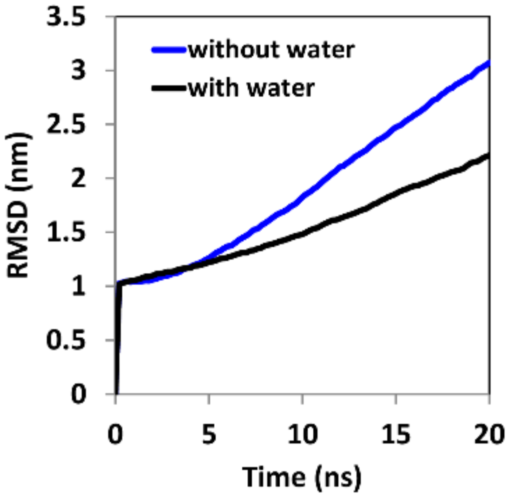

Figure S6. Root Mean Square Deviation (RMSD) graph demonstrating the diffusion behavior of CBZ with and without the presence of water. The graph shows that CBZ diffuses more effectively in the absence of water, indicated by higher RMSD values over time. This increased diffusion in a methanol-only environment contrasts with the more stable clustering observed when water is present, further highlighting the hydrophobic effect and the enhanced clustering tendency in hydrophilic solvent environments.

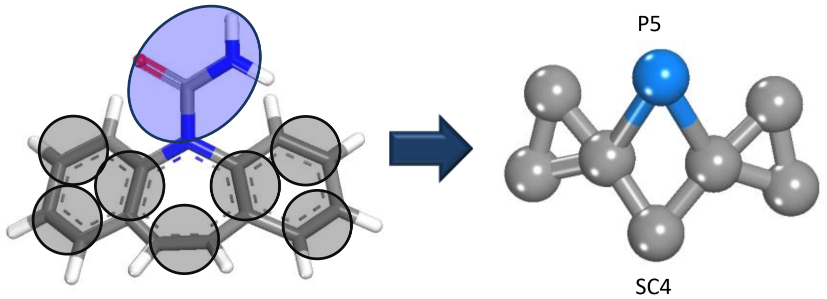

Figure S7. All atom (left) and CG model (right) for CBZ molecule. Grey, blue, red and white represent carbon, nitrogen, oxygen and hydrogen, respectively.

Table S1. Methanol to carbamazepine ratio in dense liquid clusters from different initial concentrations

Initial concentration (mg/ml)	3	5	7	9
The ratio of methanol to CBZ in the dense liquid cluster	3.88:1	2.89:1	2.69:1	2.42:1

Supporting Video 1: Whole process of the dense liquid cluster formation from 3 mg/ml of carbamazepine in pure methanol droplets. The video speeded up 12x. Scale bar: 50 µm.

Supporting Video 2: Whole process of the dense liquid cluster formation from 3 mg/ml of carbamazepine in methanol: water mixture (9:1) droplets. The video speeded up 20x. Scale bar: 50 µm.

Supporting Video 3: Whole process of the dense liquid cluster formation from 3 mg/ml of carbamazepine in methanol: water mixture (7:3) droplets. The video speeded up 20x. Scale bar: 50 µm.

Supporting Video 4: Crystallization of carbamazepine during the evaporation. The video speeded up 3x. The initial condition was 9 mg/ml carbamazepine in pure methanol droplets. Scale bar: 50 µm.

Supporting Video 5: Coalescence of the dense liquid clusters inside the droplets. The video speeded up 2x. The initial condition was 3 mg/ml of carbamazepine in pure methanol droplets. Scale bar: 50 µm.

Supporting Video 6: Crystal nucleation followed by the dissolution of the dense liquid clusters in droplets. The initial condition 3 mg/ml carbamazepine in methanol: water mixture (7:3) droplets. Scale bar: 50 µm.

Supporting Video 7: Crystal nucleation followed by the dissolution of the dense liquid clusters in droplets of high concentration. The initial condition was 9 mg/ml carbamazepine in methanol: water mixture (7:3) droplets. Scale bar: 50 µm.

Supporting Video 8: Brownian movement of the clusters inside of a droplet. The initial condition was 3 mg/ml carbamazepine in methanol: water mixture (7:3) droplets. Scale bar: 50 µm.
